# Predicting Choice Behavior of Group Members

**DOI:** 10.3389/fpsyg.2020.00508

**Published:** 2020-03-24

**Authors:** Lukas Spieß, Harold Bekkering

**Affiliations:** Donders Institute for Brain, Cognition, and Behaviour, Radboud University, Nijmegen, Netherlands

**Keywords:** group, categorization, individuation, assimilation, prediction, preferences

## Abstract

Meaningful social interactions rest upon our ability to accurately infer and predict other people’s preferences. Ireferen doing so, we can separate two sources of information: knowledge we have about the particular individual (individual knowledge) and knowledge we have about the social group to which that individual belongs (categorical knowledge). However, it is yet unclear how these two types of knowledge contribute to making predictions about other people’s choice behavior. To fill this gap, we had participants learn probabilistic preferences by predicting object choices of agents with and without a common logo printed on their shirt. The logo thereby served as a visual cue to increase perceptions of groupness. We quantified how similar predictions for a specific agent are relative to the objective individual-level preferences of that agent and how close these predictions are relative to the objective group-level preferences to which that agent belongs. We found that the logo influenced how close participants’ predictions were to the individual-level preferences of an agent relative to the preferences of the group the agent belongs to. We interpret this pattern of results as indicative of a differential weighting of individual and categorical group knowledge when making predictions about individuals that are perceived as forming a social group. The results are interpreted in an assimilation account of categorization and stress the importance of group knowledge during daily social interactions.

## Introduction

Meaningful social interactions rest upon our ability to accurately infer and predict other people’s preferences. In doing so, we can separate two sources of information: knowledge we have about the particular individual (individual knowledge) and knowledge we have about the social group to which that individual belongs (categorical knowledge). Therefore, the question arises how we subsequently weigh these two sources of knowledge when predicting individual-level behavior. Given the societal implications of inappropriate generalizations, stereotyping, and accompanying prejudices in daily social interactions, gaining more insights into the mechanisms behind the formation and use of social categorical knowledge is crucial. Hence, the current study aims to disentangle the contribution of individual-specific and social categorical knowledge when predicting people’s choice behavior.

How we process individual-specific and categorical knowledge during social interaction is poorly understood. At the individual level, observed actions are the result of a complex interplay of one’s intentions, traits, beliefs, and preferences in a given context. However, observers do not have direct access to this private information, neither is it clear how they relate to the observed behavior. Moreover, making inferences purely on the basis of individuating information is a complex and cognitively demanding process in a fast-paced social world (Macrae et al., 1994, 1999b; Sherman et al., 1998). Following Dunbar (2010), humans tend to interact with up to 150 people who are important for their daily activities. As such, relying on individual knowledge alone additionally poses an information processing bottleneck. At the categorical level, knowledge can be accumulated empirically by repeatedly observing and aggregating the actions of categorized individuals or explicitly through exposure to media or other means of social learning. This bears the question as to how we differentiate between individual-specific and categorical information and how they inform predictions we make about other people’s choice behavior.

Numerous studies have shown that perceivers categorize individuals on the basis of characteristics such as gender (Ellemers, 2018), age (Palmore, 2015), ethnicity (Dovidio et al., 1986; Priest et al., 2018), political and religious orientation (McDermott, 2009), and many more dimensions. Once an individual has been categorized into one or more context-relevant social groups, category-specific knowledge quickly becomes activated (Devine, 1989; Fiske and Neuberg, 1990). Categorical knowledge can entail information and expectations about behavioral tendencies, intentions, beliefs, preferences (Fiske and Neuberg, 1990) and affect the processing (e.g., encoding) and (mnemonic) representation of target-related information (Bodenhausen, 1988; Stangor and McMillan, 1992). As a consequence, the activation of social categorical knowledge can have powerful effects on perceivers’ evaluations and impressions toward members of that category: phenomena well-studied in the field of stereotypes and prejudices (e.g., Brigham, 1971; Brewer, 1988; Shapiro and Neuberg, 2007; Rachlinski et al., 2009; Spencer et al., 2016).

The aim of the current study is to disentangle the contribution of individual-specific and social categorical knowledge when predicting people’s choice behavior. An underlying mechanism described in the literature on categorization in general, and stereotypes in particular, is that (social) categorical knowledge can act as an interpretive frame when evaluating within-category exemplars such as members of a social group (Hilton and vonHippel, 1996; Hicklin and Wedell, 2005). As a consequence, evaluations of individual group members tend to be biased in the direction of the knowledge we have about the group as a whole – an effect generally referred to as assimilation (Rothbart et al., 1997; Hicklin and Wedell, 2005). Importantly, assimilation also implies that the perceived variability within the category or social group decreases, thereby making individual group members appear more similar to each other. Research has identified a host of factors that affect the degree to which categorical knowledge is utilized. For instance, the utilization of categorical knowledge is increased when (*i*) it is acquired through empirical observation rather than explicit instruction (Kim and Lee, 2017), (*ii*) the target member fits well the social category (Maddox, 2004; Ma et al., 2018), (*iii*) the category in itself is cohesive (Patalano and Ross, 2007; Kim and Lee, 2017), (*iv*) the category knowledge is relevant (Zukier and Pepitone, 1984), (*v*) there is uncertainty at the individual level (Locksley et al., 1982; Wichman, 2012), (*vi*) (visual) attention is directed to the social category (Macrae et al., 1999a), (*vii*) there are strong prejudices toward the category (Locke et al., 1994; Lepore and Brown, 1997) but see: (Akrami et al., 2006) and (*viii*) when the social category represents an outgroup (for a review see Rubin and Badea, 2012). In contrast, limited research has focused on investigating simultaneously how categorical and individual-specific information acquired from individual-level observations inform our predictions about people’s choice behavior. This is important as people’s behavior is typically not only driven by individual-specific beliefs and preferences but also by norms and expectations imposed by context-relevant social groups. This study investigates how we predict individual behavior by separately measuring how those predictions are affected by categorical and individual knowledge and aims to achieve a deeper understanding of the cognitive processes involved and the underlying knowledge representations at the individual and categorical level.

To this end, we designed an experiment that allowed us to quantify to what extent participants’ choice predictions are informed by individual-specific knowledge and categorical group knowledge accumulated through individual-level observations. Moreover, we investigated whether accumulated categorical knowledge will be subsequently generalized to new and unknown group members. Participants were instructed to learn probabilistic object preferences of multiple agents by predicting which of the objects the agents are most likely to choose. Perceptions of groupness were induced by printing a common logo on the shirts of some of the agents (Logo condition). The remaining agents did not have a logo printed on their shirt (No Logo condition) Preferences in both conditions were defined at two levels: At the individual level, preferences for each agent were defined using individual-specific preference distributions. At the group level, preferences were defined as the average of all individual-specific preference distributions within a condition. Using a measure from information theory, we quantified how similar participants’ predictions in the Logo and No Logo conditions are to their respective individual-level and group-level preferences. We hypothesized that relative to the No Logo condition, participants’ predictions in the Logo condition will become more similar to the group-level preferences while less similar to the individual-level preference. We will take this as evidence that predictions for categorized individuals are marked by an increase in the use of categorical group knowledge but a decrease in the use of individual-specific knowledge. Moreover, we expect that the generalization of the accumulated categorical group knowledge to a new agent would be stronger in the Logo as compared to the No Logo condition.

## Materials and Methods

### Participants

A total of 26 participants (18 females; *M*_age_ = 23.42 years, *SD*_age_ = 3.45 years, range 18–30 years) took part in the current study. Participants were recruited through the Radboud University online research registration system and none of them reported a history of neurological or psychiatric disorder. The study was approved by the local ethics committee (ECG2010-0910-058) and all participants gave written informed consent according to the declaration of Helsinki. Participants received 10€ or an equivalent of 1 course credit as compensation.

### Stimulus Material

**FIGURE 1:**
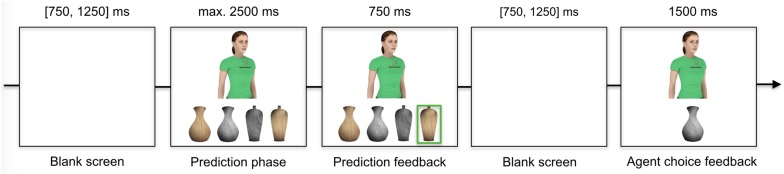
Proceedings of a trial.

All stimuli were presented on an LCD monitor (Benq XL2420Z, 24 inches, 120 Hz, 1680 × 1050 pixels) connected to a stationary computer (Dell Precision T3610, 4 × 3.7 GHz, 8GB ram) running Windows 7. The stimulus material consisted of ten customized computer-generated agents, a custom-designed logo and four custom-designed vases designed that served as the choice objects (see Figure 1). The agents (50% female) were portrayed from their hips upward in a frontal upright position with a resolution of 384 × 384 pixels. To increase visual discrimination of same-sex agents, half of them were designed with blond hair color, the remaining half with brown hair color. Hair color was further randomly combined with hairstyle (female: long smooth hair vs. short tail; male: wavy vs. fringe). The clothing of agents only differed in terms of the color of the shirt and whether or not the shirt had a logo printed on it. The logo was superimposed on the upper left part of the shirt such that it could be easily seen. The logo displayed an abstract figure in a sporty posture with the text “Sutternden.” Both the design and text of the logo were chosen to have no meaning or natural association with any agent or task characteristic.

The choice objects consisted of four self-designed vases that differed with regard to color (stone-like texture vs. wood-like texture) and shape (pear-shaped vs. cone-shaped). Counterbalancing the color and shape dimensions yielded four unique vases. Choice objects were presented with a resolution of 106 × 175 pixels.

### Task Design and Procedure

Participants were told that there are eight different agents and that each agent prefers one of the four different vases. Participants were instructed to give predictions about the most likely choice of a given agent per trial. They were also informed that preferences are not absolute and that every agent will eventually choose each object at least once. No information was given about the logos or any type of group membership. Participants had to use four fingers of their dominant hand to indicate their prediction on a time accurate (1 ms) in-house button box. The order of the choice objects on the screen corresponded with the horizontal alignment of the response keys. The experiment started with a written repetition of the instructions.

#### Block 1

The first block consisted of 400 trials divided into 4 sub-blocks of 100 trials separated by short breaks. Every trial (see Figure 1) started with a blank screen presented for a variable time (between 750 ms and 1250 ms, *M* = 1000 ms) followed by a prediction phase with a timeout of 2500 ms. During the prediction phase, the agent was presented on top of the four choice objects. To highlight the participant’s predictions (prediction feedback), a green square appeared around the selected vase for 750 ms followed by a blank screen (presented between 750 ms and 1250 ms, *M* = 1000 ms). The trial ended with the presentation of the agent alongside the agent’s actual choice for 1500 ms (agent choice feedback).

In Block 1, eight unique agents were each presented 50 times in a randomized order. Half of the agents had a logo printed on their shirt (Group condition) while the other half did not (Individual condition). Moreover, and independent of whether a logo was printed on an agent’s shirt, each agent had a unique shirt color. This was done so that participants could easily distinguish among individual agents. Across participants, agents were randomly assigned to the Logo and No Logo condition with the constraint that in each condition half of the agents were female and half were male. From a physical stimulus perspective, the only difference between the Logo and No Logo condition was whether or not a logo is printed on the shirts of the agents. The left-to-right order of the displayed choice objects was randomized across trials and participants.

**FIGURE 2:**
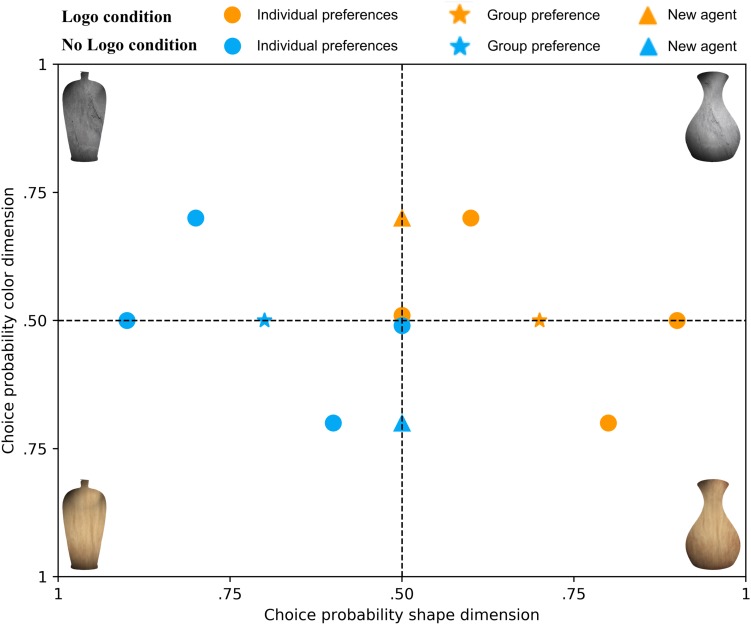
Experimentally manipulated individual-level and group-level preferences. Presented are the experimentally defined individual agent preference distributions (filled circles) belonging to the Logo and No Logo condition (orange and blue, respectively). The group-level preference distributions are depicted by the asterisks, which is the average of all individual preference distributions in that condition. The newly introduced agents in Block 2 are depicted by triangles. Vases varied on the shape and color dimensions. The x-axis reflects the probability of choosing a certain color and the y-axis a certain shape, resulting in preferences for the four choice objects.

Agent preferences for the choice objects were defined in terms of categorical probability distributions at two levels: Individual level and group level. The average over the individual-level distributions for the Logo and No Logo condition, respectively, defined the group-level preference distributions. Importantly, the individual-level distributions of the two conditions were symmetric with respect to each other (see Figure 2). That is, if an agent in the Logo condition preferred the wooden cone-shaped vase, there was also an agent in the No Logo condition that equally preferred the stone-like pear-shaped vase. The symmetrical preference distributions at the individual level ensured that preferences at the group level were symmetric as well. Moreover, preferences at the individual level within each condition were symmetrically distributed around their respective group-level preference distributions.

In each condition, two agents had a preference for both dimensions of the choice objects (i.e., a particular shape and a particular color), only one agent had a preference for a single dimension (e.g., only a particular shape), and one agent did not have a preference at all (i.e., uniform distribution over shape and color). Due to the symmetrical arrangement of the individual-level distributions within and across the Logo and No Logo conditions, group-level preferences only existed along a single dimension. The assignment of the group-level preference dimension (color or shape), as well as the specific phenotype (i.e., the particular color or shape), was counterbalanced across participants. Overall, every choice object was chosen for an equal number of times.

### Block 2

In Block 2, participants were told that two new agents will be added and that they should try to learn their preferences as well. No further information was given. Of the 100 trials that participants had to complete in the second block, 60 trials showed one of the two new agents (30 times per condition). The remaining eight agents were evenly distributed across the remaining 40 trials. Crucially, the newly introduced agents did not have a preference on the same dimension that was also preferred by the two groups (e.g., shape); instead, they only had a preference along the dimension that was not preferred by the groups (e.g., color; see Figure 2). In other words, the new agents only exhibited an individual-level preference. This would allow us to investigate to what extent participants learn individual-level preferences that are independent of group-level preferences. The preferences of the eight agents of Block 1 did not change in Block 2.

In total, the experiment lasted on average 55 min. After completion, participants were asked whether they got the impression that the two types of agents (with and without a logo) formed two groups that each had a preference for a particular shape or color or the vases.

To our surprise, only a single participant reported that there were certain patterns in the preferences of individual agents, pointing to how subtle the manipulation apparently was.

### Data Analysis and Design

A full within-subject design was used. As dependent variable, we employed a measure referred to as the Kullback-Leibler divergence (KL_*Div*_; Kullback and Leibler, 1951). The KL_*Div*_, also called relative entropy, measures the directed divergence from one probability distribution to another and is here reported in units of bits. Technically, it quantifies how much information is lost when a distribution Q is used to approximate a distribution P and is consequently not symmetric. Within the Bayesian framework, it can be interpreted as the information gain when one’s prior distribution Q is updated to the posterior distribution P. Importantly, in order to be able to calculate the KL_*Div*_ from Q to P, the support of distribution Q has to be a subset of the support of distribution P. In other words, both probability distributions need to have non-zero probabilities over the same set of categories. This was not the case when, for example, a participant never predicted an agent to choose a particular choice object although the agent actually did choose each choice object at least once. This happened in 13 participants for at least one agent. In these cases, before transforming participants’ predictions into separate probability distributions for each agent (prediction distribution), additive smoothing was applied by adding one pseudocount over the entire prediction distribution’s support of an agent.

Subsequently, KL_*Div*_ was used to independently quantify how much participants’ predictions diverge from the individual-level preference distribution (Individual level) and the respective group-level preference distribution (Group level). At the individual level, we calculated for every agent the KL_*Div*_ from the prediction distribution of that agent to the agent’s actual preference distribution. The data was then averaged across the four agents separately for the Group and Individual condition. A small KL_*Div*_ value at the individual level indicates that participants’ predictions were on average similar to the true preferences of the respective agents. At the group level, we calculated how much the predictions for each agent diverged from the group-level preferences. The data was then averaged across the four agents separately for the Group and Individual condition. A small KL_*Div*_ value here indicates that participants’ predictions for individual agents were on average very similar to the average true preference of all agents within the respective condition.

In order to answer our primary research question, we analyzed the data from Block 1 and 2 separately but in the same way. For Block 1 and Block 2, The KL_*Div*_ values were subjected to a two-way repeated measures ANOVA with factors Condition (Logo vs. No Logo) and Levels (Individual preference vs. Group preference). The effect of interest pertains to the Condition by Level interaction effect. Interaction effects were further analyzed with paired-sample *t*-tests. Unless otherwise specified, all *p*-values were two-tailed.

In an assimilation analysis, we further investigated how differentiated participants’ predictions for the four agents in each condition were as a function of time (i.e., trials). This represents a direct test of assimilation. To do so, we first calculated the average distribution of participants’ predictions across the four agents separately for each trial, for each participant, and for each condition. We then used a symmetrical KL_*Div*_ (Johnson and Sinanovic, 2001) to quantify how similar the predictions for each agent are relative to the average distribution of participants’ predictions that we calculated in the first step. In order to account for zero-values in the distributions, we smoothed all prediction distributions by adding a pseudocount over the entire distribution’s support. The resulting divergence values were then averaged to a single value per trial, participant, and condition reflecting the average statistical divergence from the mean distribution in bits. This is similar to what is known as the mean absolute deviation. This analysis permits us to investigate how similar participants’ predictions for the four agents in each condition are over time, regardless of how accurately one has learned the objectively defined individual-level and group-level preferences. The assimilation analysis was done on the Block 1 data only.

## Results

### Block 1

**FIGURE 3:**
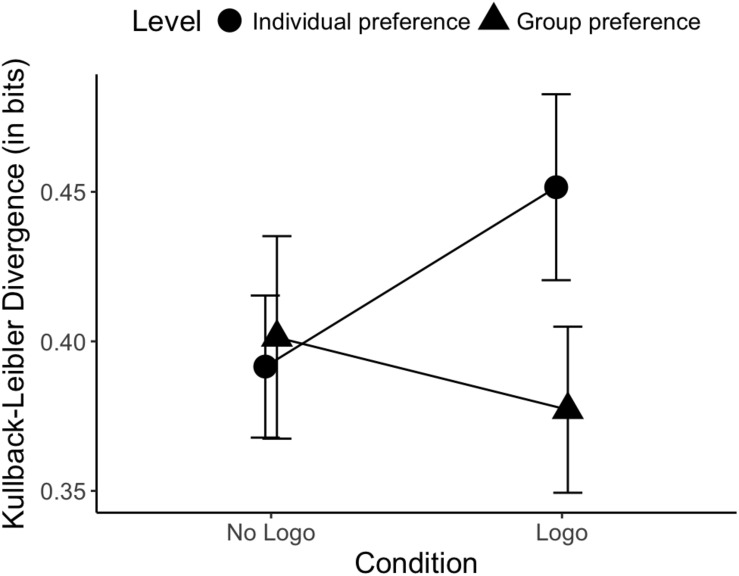
Level by Condition interaction on the Kullback-Leibler divergence for Block 1. The predictions in the Logo condition relative to the No Logo condition are more similar to the group-level preferences (have a smaller KL divergence) while less similar to the individual-level preferences. Error bars represent standard error of the mean.

On average, participants missed 2.17% of the trials. The repeated measures ANOVA revealed no main effect for Level [*F*(1, 25) = 3.54, *p* = 0.071] or Condition [*F*(1, 25) = 0.15, *p* = 0.70]. However, as can be seen in Figure 3, a significant Condition by Level interaction was found [*F*(1, 25) = 9.12, *p* = 0.006, η*_*p*_*^2^ = 0.27]. This indicates that the KL divergence from the prediction distributions to the individual-level and group-level preference distributions differed depending on whether or not the agents had a logo printed on their shirt. Inspection of the individual cell means suggests that the Logo condition is marked by an increased KL_*Div*_ for the individual-level preference distributions (*M*_Logo_ = 0.45 bits, *SD*_Logo_ = 0.16 bits vs. *M*_No Logo_ = 0.39 bits, *SD*_No Logo_ = 0.15 bits) but a decreased KL_*Div*_ for the group-level preference distributions (*M*_Logo_ = 0.38 bits, *SD*_Logo_ = 0.14 bits vs. *M*_No Logo_ = 0.40 bits, *SD*_No Logo_ = 0.17 bits). *Post hoc* analyses within each Level, however, show that these effects are not statistically significant on their own [Individual level: *t*(25) = 1.34, *p* = 0.19; Group level: *t*(25) = -0.48, *p* = 0.64].

### Block 2

Participants missed on average 0.88% of the trials. The repeated measures ANOVA revealed a significant main effect of Measure [*F*(1, 25) = 32.45, *p* < 0.001, η*_*p*_*^2^ = 0.56] with the KL divergence being larger for the Group level (*M* = 0.81, *SD* = 0.40) than the Individual level (*M* = 0.52, *SD* = 0.32). Neither the main effect of Condition [*F*(1, 25) = 0.08, *p* = 0.77] nor the Condition by Level interaction was significant [*F*(1, 25) = 1.69, *p* = 0.21].

### Assimilation Analysis

**FIGURE 4:**
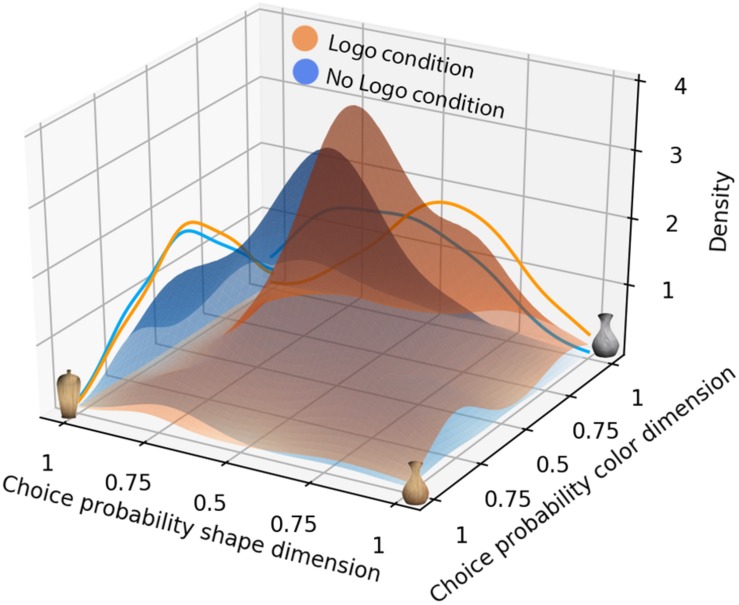
Gaussian densities of participants’ predictions in Block 1. The estimated Gaussian density functions represent the bivariate (combined color and shape) prediction distributions for all agents and all participants for the No Logo condition (blue) and Logo condition (orange) of Block 1. The curves on the left and right walls depict the univariate distributions for the color and shape dimensions, respectively. Notice the higher peak in the bivariate Gaussians for the Logo condition relative to the No Logo condition.

In order to visualize the distribution of participants’ predictions across the Logo and No Logo condition, bivariate and univariate Gaussian kernel density functions were estimated. Scott’s rule (Scott, 2015) was used to select appropriate bandwidths for the bivariate and univariate distributions. The resulting Gaussian density functions are shown in Figure 4. From there it can be seen that prediction distributions for agents belonging to the Logo condition are more densely clustered compared to the No Logo condition. Visual inspection of the marginal distributions depicted on the site walls also suggests that the denser clustering in the Logo condition is mostly due to smaller variability when predicting the dimension that is also preferred by the group – here the shape dimension.

In a first analysis, we tested whether predictions for agents in the Logo condition are more similar to each other than predictions in the No Logo condition. For the purpose of establishing a general assimilation effect, we consider the prediction distributions of the last trial as it contains the information of all preceding trials. Importantly, visual inspection and formal tests revealed right-skewed distribution of KL_Div_ values rendering paired-sample t-tests inappropriate. Consequently, we used a non-parametric paired-sample Wilcoxon signed-rank test. A Wilcoxon signed-rank test on the average KL_Div_ between the agents on the last trial shows that the KL_Div_ was significantly smaller in the Logo condition (*Mdn* = 0.2) compared to the No Logo condition (*Mdn* = 0.29), *Z* = -2.49, *p* = 0.013). This indicates that the predictions for the different agents in the Logo condition were more similar to each other than they were in the No Logo condition.

**FIGURE 5:**
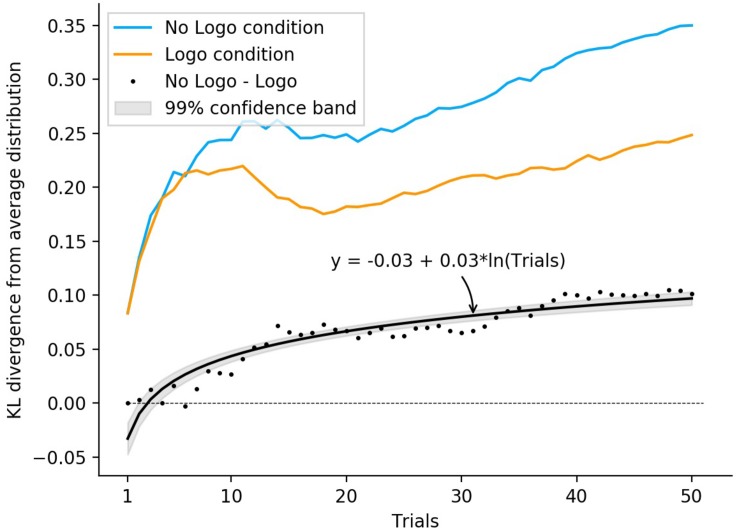
Time course of the assimilation effect in Block 1. The upper lines depict the average Kullback-Leibler divergence for the Logo and No Logo conditions in Block 1, respectively, in orange and blue. Black dots represent the mean difference between the Logo and No Logo condition, with the black curve showing a fitted logarithmic regression model with its 99% confidence band. It is clearly visible that the average KL-divergence is at almost any point descriptively smaller in Logo condition compared to the No Logo condition. This suggests that participants’ predictions for the four agents in the Logo condition are less differentiated relative to the No Logo condition.

To investigate the time course of this effect, we calculated the average difference between the Logo and No Logo condition across participants for each trial and fitted a logarithmic regression on the event series data (see Figure 5). Overall, the regression model was highly significant [*F*(1, 48) = 55.39, *p* < 0.001, *R*^2^ = 0.53] and yielded a significant coefficient for trial (β = 0.02, *t* = 7.44, *p* < 0.001), which indicates that the difference across the two conditions increased logarithmically over time.

## Discussion

In the present experiment we investigated to what degree predictions about other people’s choice behavior are differentially informed by knowledge that we have about preferences at an individual level and knowledge that we have about preferences at a categorical group level. In addition, we also examined whether accumulated categorical group knowledge would be generalized onto newly introduced individuals. In our task, participants had to learn probabilistic preferences of agents that either had a common logo printed on their shirt to induce strong perceptions of social groupness (Logo condition) or not (No Logo condition). We then quantified how similar the predictions for a specific agent are relative to the objective individual-level preferences of that agent and how close these predictions are relative to the objective preferences of the group to which that agent belonged. We found that our logo manipulation influenced the degree to which predictions resembled the individual-level preferences of an agent relative to the preferences of the entire group the agent belongs to. Our main finding thus suggests that stronger perceptions of groupness can lead to a differential weighting of individual and categorical group knowledge when making predictions about other peoples’ choice behavior. In particular, our findings suggest that increased perceptions of groupness can lead to an increased use of categorical group knowledge relative to the use of individual-specific knowledge. Note that this occurred even though the logo that served to induce perceptions of groupness had no predictive value in itself. The logo only served as a visual cue that should induce stronger perceptions of groupness relative to not having a logo printed on one’s shirt. Therefore, the preference distributions of agents with a logo on their shirt were essentially the same but mirrored compared to the preference distributions of agents without a logo on their shirt. Whether or not an agent had a logo on his or her shirt was therefore the only systematic difference between the Logo and No Logo condition. In line with the literature, we interpret the findings in an assimilation account (Rothbart et al., 1997; Hicklin and Wedell, 2005), wherein evaluations of individual group members tend to be biased in the direction of our categorical knowledge of the group. Importantly though, assimilation can be realized in different ways.

One possibility is that participants detected the logo on the shirts of some agents and inferred from this that they probably form a social group. Due to this, participants expect agents within that social group to share more characteristics (i.e., preferences) among each other compared to agents that are not perceived to form a group. The consequence is that predictions for individual group members are then influenced by the knowledge about all other group members. Alternatively, the logo manipulation might have drawn participants’ visual attention to the logo, such that the actual agent choices were not only associated with the particular individual in terms of gender, face, and hair-style, but also with the perceptual logo itself. When predictions had to be made, those predictions are then not only informed by the knowledge associated with that individual but also by the accumulated knowledge across all individuals associated with the perceptual logo itself. This is similar to how perceptual cues related to age, gender, ethnicity or clothing that, upon encounter, can trigger associations and expectations related to the characteristic social variables such as traits, beliefs, and desires.

Regardless of the underlying mechanism, two major consequences emerge. For one, predictions for individual group members are biased toward the average preference of all group members. For another, individual group members’ preferences are perceived to be more similar to each other than they actually are. Both effects are indicative of assimilation and are frequently found in studies on categorization and stereotyping (Rothbart et al., 1997; Hicklin and Wedell, 2005). Our findings are compatible with the idea that assimilation was stronger in the Logo condition than in the No Logo condition. This is further substantiated by the assimilation analysis providing clear evidence that participants’ predictions for individual agents in the Logo condition are more similar to each other compared to the No Logo condition. This results in predictions for agents in the Logo condition that are less individual-specific, thereby leading to a relative (though not significant on its own) decrease in the utilization of individual-specific knowledge. As stated before, assimilation can in principle be based on any value and does not necessarily have to bias within-category estimates toward an objective category mean, such as the group-level preferences that we have experimentally created in the current study. Although descriptively we did find a relative increase in the utilization of group knowledge in the Logo condition, *post hoc* tests revealed that this effect on its own was not statistically significant. A possible explanation for this is that assimilation did not sufficiently bias individual agent representations toward the objective group-level preference, but rather toward the subjectively inferred group-level preferences. In other words, participants clustered the agents in the Logo condition around what they considered to be the average agent preference rather than what the actual average agent preference really was, namely the experimentally defined group-level preference. This might have happened because the task was designed in such a way that the actual group-level preferences had to be inferred by aggregating the individual-level preferences. As a consequence, precise knowledge about the actual group-level preference requires a sufficiently large number of observations at the individual level and hence accumulates only slowly over time.

Interestingly, we did not find that participants generalize their group knowledge to a different degree depending on whether the newly introduced and unknown agent belongs to the Logo or the No Logo condition. This contrasts with earlier findings that generalization of categorical group knowledge does take place, particularly when the group member is prototypical for the group (e.g., Maddox, 2004; Ma et al., 2018). Although we can only speculate about the lack of an effect here, it might be that participants’ representations of the group-level preference in the Logo condition were not distinct enough to see clear generalization effects. Although descriptively we did find a relative increase in the utilization of group knowledge in the Logo condition in Block 1, the *post hoc* analysis revealed that this effect on its own was not statistically significant. Alternatively, participants may not have been sufficiently aware of the fact that one of the newly introduced members also had a logo printed on his/her shirt, which decreases the chance of generalizing categorical group knowledge.

Nevertheless, the current study design bears the potential for novel and important insights in relation to categorization and assimilation. In contrast to most other studies, we simultaneously focused on the processing of categorical and individual knowledge and compared how it is affected by whether predictions had to be made for individuals that are part of a social group or not. Interestingly, participants did not simply group the agents into equal dichotomous categories based on the presence (or absence) of the logo nor were agents grouped in terms of their (common) preferences. Instead, participants seemed have categorized or grouped the agents with a logo on their shirt more so than the agents without a logo. This is corroborated by the assimilation analysis revealing that preferences of agents with a logo are perceived to be more similar to each other than preferences of agents without a logo. Moreover, the current experiment was introduced without ever mentioning anything about groups or logos and had to be performed exclusively on an individual level (i.e., predict individual agent choices). The fact that only a single participant has realized the presence of group-level preferences not only shows how efficient our subtle logo manipulation was, it also suggests that it affected participants’ predictions unintentionally and potentially even outside their awareness. Contrary to the majority of studies in which pre-existing stereotypes were used, our study required participants to learn the group-level preferences from observations at an individual level. This gave us direct control over the content of the categorical knowledge and allowed us to investigate not only the degree to which categorical knowledge is used but also how it is formed in the process of learning individual-specific information.

Although this study deviates from the literature in many ways, and is in that sense explorative by nature, our findings could have potentially important implications for the emergence of stereotypes in naturalistic social groups. The results suggest that a subtle and arbitrary perceptual cue such as a common logo is sufficient to trigger basic categorization-related information processing mechanisms (e.g., assimilation) that affect how we are going to relate, accumulate, and weigh perceived individual-level behavior in relation to the person displaying that behavior and the social group to which he or she belongs. This could facilitate the formation of stereotypes in two interrelated ways: First, categorization decreases the extent to which we interpret subsequent individual-level behavior as reflecting unique characteristics of the individual. This holds even if the observed behavior is non-diagnostic for group membership. Second, it increases the extent to which we interpret the observed behavior in light of what we know about the group as a whole. As a consequence, categorized individuals are perceived to be less unique and are expected to behave in accordance with what we know about the group. Future studies could build upon the current work by investigating the use of individual and categorical knowledge in in-group/out-group scenarios (Rubin and Badea, 2012) and could study the interdependent dynamics of how updated knowledge on a group level affects representations at the individual level and vice versa. Moreover, the current study addresses categorization in only one way, namely categorization on the basis of visual features that allow for extracting information that relate to, for example, ethnicity, age, gender, jobs (e.g., uniforms), and sport teams (e.g., tricots). However, we also categorize individuals around us in terms of whether they are our friends, family members, colleagues or even strangers. In these cases, categorization may not be based on perceptual cues but rather abstract features coupled to semantic knowledge and episodic memory. Studying these more abstract instances of categorization opens promising avenues for future research.

## Conclusion

To conclude, we have provided evidence that categorical and individual-specific knowledge differentially contribute to making predictions about other people’s choice behavior depending on the degree to which they are perceived to belong to a social group. Increased perceptions of groupness can lead to an increased use of categorical group knowledge relative to the use of individual-specific knowledge. As a consequence, evaluations of individual group members tend to be biased in the direction of the group – an effect generally referred to as assimilation. In line with this, strongly perceiving other people as forming a social group also leads to more similar predictions about their choice behavior.

## Data Availability Statement

The datasets generated for this study are available on request to the corresponding author.

## Ethics Statement

The studies involving human participants were reviewed and approved by Ethics Committee of the Faculty of Social Sciences (ECSW), Radboud University Nijmegen. The patients/participants provided their written informed consent to participate in this study.

## Author Contributions

LS contributed to all aspects of the research process. HB contributed to designing the study, interpreting the results, and reviewing the manuscript.

## Conflict of Interest

The authors declare that the research was conducted in the absence of any commercial or financial relationships that could be construed as a potential conflict of interest.
